# Risk Factors Contributing to Adhesive Disease Following Primary Cesarean Section: A Cross-Sectional Study

**DOI:** 10.7759/cureus.95708

**Published:** 2025-10-29

**Authors:** Rajlakshmi Mehanathan, Vidhya Selvam, M Pavithra, Niveditha Prasath

**Affiliations:** 1 Obstetrics and Gynecology, Sree Balaji Medical College and Hospital, Chennai, IND

**Keywords:** adhesions, cesarean section (c-section), intra- abdominal surgery, post-operative morbidity, risk factor approach

## Abstract

Background: Cesarean section is one of the most commonly performed obstetric surgeries worldwide, with rising rates of repeat procedures. Intra-abdominal adhesions are a frequent complication following prior cesarean deliveries and can complicate subsequent surgeries, leading to increased operative time, blood loss, and risk of visceral injury. Understanding the distribution, severity, and risk factors for adhesion formation is crucial for improving surgical outcomes. This study aimed to evaluate the distributive pattern of adhesions in women undergoing repeat cesarean section, identify associated demographic and clinical risk factors, and assess the impact of adhesions on operative parameters and postoperative outcomes.

Methods: A prospective, observational cross-sectional study was conducted at Sree Balaji Medical College and Hospital, Chennai, including 104 women scheduled for repeat cesarean delivery. Adhesions were assessed intraoperatively based on site, severity, and size using the Tulandi and Lyell composite adhesion scoring system. Maternal demographic and clinical data, including age, socioeconomic status, comorbidities, type, and indication of previous cesarean, were recorded. Operative outcomes, such as duration of surgery, estimated blood loss, and postoperative hospital stay, were analyzed. Statistical analysis included chi-square tests for categorical variables and independent t-tests for continuous variables, with p < 0.05 considered significant.

Results: Adhesions were present in 31.7% (33/104) of participants. Emergency cesarean, low socioeconomic status, gestational diabetes, and certain previous CS indications (cervical dystocia, second stage arrest, failed induction with PROM) were significantly associated with adhesion formation (p < 0.05). Women with adhesions experienced significantly higher mean blood loss (490.3 ± 127.4 vs 433.9 ± 120.6 mL, p = 0.03) and longer operative time (67.3 ± 10.9 vs 45.9 ± 7.6 min, p < 0.001).

Conclusion: Intra-abdominal adhesions are common in repeat cesarean sections, particularly following emergency procedures and in women with specific comorbidities. Adhesions significantly impact surgical outcomes, highlighting the importance of careful surgical planning and preventive strategies in women undergoing repeat cesarean deliveries.

## Introduction

Cesarean section is a vital surgical procedure in modern obstetrics that has significantly contributed to the reduction of maternal and perinatal mortality. Initially performed only in life-threatening situations where vaginal delivery was unsafe for the mother or fetus, the cesarean section has evolved into one of the most frequently performed surgical procedures worldwide [1]. Its judicious use has saved countless lives, particularly in cases of obstructed labor, malpresentation, fetal distress, or placenta previa [2]. However, in recent decades, the rate of cesarean deliveries has risen far beyond what can be explained by medical necessity, generating global concern regarding its overuse and long-term consequences [3].

While the cesarean section is often life-saving, its increasing frequency - especially of repeat procedures - has introduced a spectrum of postoperative complications. One of the most prevalent yet under-recognized among these is the development of intra-abdominal adhesions [4]. Adhesions are fibrous bands of connective tissue that form between internal organs or between organs and the abdominal wall following surgery or infection. They arise as part of the normal healing process after tissue trauma, but can become pathological when they distort normal anatomy [5]. In obstetric and gynecologic practice, adhesions after cesarean delivery can cause a variety of complications, including chronic pelvic pain, infertility, bowel obstruction, and increased operative difficulty in subsequent surgeries [6].

From a surgical perspective, dense adhesions can complicate future abdominal operations by obscuring anatomical landmarks, prolonging operative time, and increasing the risk of intraoperative injuries to the bladder, bowel, or uterus [7]. Furthermore, adhesion formation contributes to greater blood loss, difficult fetal extraction during repeat cesarean deliveries, and extended postoperative recovery periods [8]. These outcomes not only elevate surgical risk but also impose a substantial burden on healthcare systems, especially in regions where cesarean deliveries are becoming increasingly common. Over the past two decades, cesarean section rates have risen sharply across the globe. In 2000, the global average was estimated at around 12%, which has since increased to over 21% by 2021 [9]. A major global analysis* *projected that, if current trends continue, nearly 29% of all births worldwide will be by cesarean section by 2030 - equating to nearly 39 million procedures annually [10].

This trend, however, shows striking regional variations. While cesarean rates exceed 40% in some countries of Latin America, the Middle East, and Eastern Europe, sub-Saharan Africa continues to report rates below 10%, largely due to limited access to surgical and obstetric facilities [9]. Although the rising rates in some low- and middle-income countries partly reflect improved access to institutional deliveries, they also point to an alarming increase in non-medically indicated cesarean sections [1].

Several interrelated factors are responsible for this global escalation. Improved access to institutional births, better availability of surgical infrastructure, and changing maternal demographics have all contributed to the rise [2,3]. In addition, non-clinical drivers, such as physician convenience, defensive medical practices due to fear of litigation, and maternal preference for elective cesarean deliveries, play a considerable role [4]. Societal perceptions that cesarean delivery is safer or more modern than vaginal delivery also influence maternal choices, particularly in urban and private healthcare settings [5].

In India, as in many developing countries, a combination of institutional practices and maternal preference has led to a steady increase in cesarean section rates. Declining practice of vaginal birth after cesarean (VBAC), increasing maternal age, and lack of awareness about the potential complications of repeat cesarean deliveries further amplify this problem [6]. A recent study conducted at a rural tertiary care center reported a high prevalence of dense intra-abdominal adhesions among women undergoing repeat cesarean sections, particularly in those with a prior emergency procedure, highlighting the rising postoperative burden [7].

Although cesarean delivery remains indispensable when medically justified, its overuse has important maternal health implications. Repeat cesarean sections significantly increase the risk of placenta previa, placenta accreta spectrum disorders, uterine rupture, and severe intraoperative hemorrhage [8]. Intra-abdominal adhesions, in particular, not only complicate repeat surgeries but also contribute to long-term morbidity through pain, infertility, and intestinal obstruction [9].

The formation of adhesions after cesarean section represents a preventable cause of surgical difficulty and maternal morbidity. Awareness of this risk underscores the importance of adhering strictly to evidence-based indications for cesarean delivery and promoting safe alternatives such as trial of labor after cesarean (TOLAC) where feasible [10]. Reducing unnecessary cesarean sections through patient education, adherence to clinical guidelines, and institutional monitoring of indications can help limit adhesion-related complications and improve overall maternal outcomes. In conclusion, while cesarean section remains one of the cornerstones of modern obstetric practice, its increasing frequency - driven by both medical and non-medical factors - poses significant challenges for maternal health. The present study aims to investigate the distributive pattern of intra-abdominal adhesions among women undergoing repeat cesarean sections and to identify the associated risk factors contributing to their development. 

## Materials and methods

Study design

The present investigation was designed as a single-centre, observational cross-sectional study aimed at assessing the pattern of intra-abdominal adhesions and identifying their associated risk factors in women undergoing repeat cesarean section. A prospective design was chosen to enable real-time documentation of intraoperative findings and minimize recall bias, while the observational approach allowed for the inclusion of naturally occurring variations in surgical presentations without experimental manipulation. The cross-sectional nature of the study provided a snapshot of adhesion prevalence and its relationship with patient characteristics and surgical outcomes at a specific point in time, thereby ensuring comprehensive assessment within the study duration.

Study setting and duration

The study was conducted in the Department of Obstetrics and Gynaecology at Sree Balaji Medical College and Hospital, Chennai, a tertiary care teaching institution with a high obstetric caseload, making it an ideal setting for research on repeat cesarean deliveries. The hospital serves a diverse patient population from both urban and semi-urban regions, providing a representative sample of women undergoing cesarean section in South India. Data collection was carried out over a one-year period, from 2024 to 2025, allowing adequate time for participant recruitment, surgical observation, and data validation. All eligible cases that met the inclusion criteria during this period were consecutively enrolled to ensure a robust dataset for analysis.

Study population

The study population comprised 104 pregnant women scheduled for repeat cesarean section during the study period. Only those who had undergone one or more prior cesarean deliveries were considered eligible for inclusion. The selection of this specific population was based on the objective of understanding adhesion formation as a postoperative sequela unique to repeat cesarean sections. These participants represented a range of obstetric profiles, including both elective and emergency procedures, thereby reflecting the true clinical variability encountered in obstetric practice.

Inclusion and exclusion criteria

The inclusion criteria encompassed all women undergoing repeat cesarean delivery who had a history of one or more prior cesarean sections. These participants were recruited irrespective of parity, socioeconomic status, or indication for surgery, provided that they consented to participate. The exclusion criteria were meticulously defined to eliminate potential confounders. Additionally, patients with a history of any other abdominal surgeries apart from cesarean section were excluded to ensure that the adhesions assessed were attributable solely to previous cesarean deliveries and not to other abdominal interventions such as appendectomy, laparotomy, or myomectomy. This exclusion strengthened the internal validity of the findings by reducing external sources of bias in adhesion formation.

Sample size determination

The sample size was estimated using Cochran’s formula for sample size determination with a 95% confidence level (1.96), with the expected proportion of the outcome in the population based on previous literature (7%) [11], and the allowable error margin (5%). Accordingly, the minimum required sample size was calculated as 104 participants, all of whom were recruited to achieve the desired level of statistical precision and representativeness.

Sampling method

A non-probability convenience sampling method was employed to enroll participants who met the eligibility criteria and consented to participate during the study period. This approach was chosen for practical feasibility, considering the hospital-based nature of the research and the limited timeframe. Although convenience sampling may have inherent selection bias, it was mitigated by the inclusion of consecutive eligible participants and adherence to standardized intraoperative assessment protocols.

Data collection protocol

Prior to initiation, the study protocol received approval from the Institutional Human Ethics Committee of Sree Balaji Medical College and Hospital (Approval No: 002/SBMCH/IHEC/2023/2071). After obtaining informed written consent from each participant, data collection commenced using a structured proforma designed to capture relevant demographic, obstetric, and intraoperative details.

Maternal characteristics such as age, parity, socioeconomic status based on the Modified BG Prasad Scale [12], and medical comorbidities (including anemia, gestational diabetes mellitus (GDM), preeclampsia, and hypothyroidism) were documented. Information on the number and nature of previous cesarean sections (elective or emergency), the indication for the prior procedures, and the interval between consecutive cesarean deliveries was also recorded. These variables were considered critical for identifying potential predictors of adhesion formation. During the repeat cesarean section, a standardized intraoperative assessment was performed by the operating surgeon. The peritoneal cavity was inspected for the presence of adhesions immediately after the incision of the abdominal wall and entry into the peritoneum. The adhesions were systematically evaluated in terms of site, type, and extent. Anatomical site classification included adhesions between the uterus and bladder, uterus and omentum, omentum and abdominal wall or fascia, and adhesions involving the bowel. Type classification differentiated between flimsy (easily separable) and dense (fibrous and vascular) adhesions. Extent was estimated based on the area involved, with dense adhesions larger than 6 cm recorded as extensive.

The Tulandi and Lyell composite adhesion scoring system was employed to grade adhesion severity objectively, allowing for reproducibility and comparison with existing literature [13]. Operative parameters such as total duration of surgery, estimated blood loss, difficulty in fetal extraction, and intraoperative complications, including bladder or bowel injuries, were carefully documented. Postoperative outcomes, including wound healing and infection, were also noted when applicable.

Statistical analysis

Data were entered into Microsoft Excel and analyzed using IBM SPSS Statistics version 26.0 (IBM Corp., Armonk, NY). Descriptive statistics were used to summarize participant characteristics and study findings. Categorical variables (such as age group, socioeconomic class, type of prior cesarean, and presence or absence of medical comorbidities) were presented as frequencies and percentages. Continuous variables, including operative time and estimated blood loss, were summarized using means and standard deviations (SD).

The chi-square test or Fisher’s exact test was applied to examine associations between categorical risk factors and the presence of adhesions, depending on expected cell counts. For continuous variables, group comparisons between women with and without adhesions were conducted using the independent t-test. A p-value of less than 0.05 was considered statistically significant. Confidence intervals (95% CI) were calculated where appropriate to estimate the precision of the observed associations.

Ethical considerations

The study was conducted in accordance with the ethical principles outlined in the Declaration of Helsinki (2013 revision). All participants were informed about the purpose and procedures of the study in their preferred language, and written informed consent was obtained prior to inclusion. Participation was entirely voluntary, and participants were assured that their decision to withdraw at any point would not affect the quality of their medical care. Confidentiality of patient data was strictly maintained throughout the study. Ethical approval was obtained from the Institutional Human Ethics Committee of Sree Balaji Medical College and Hospital, and the study adhered to all institutional and national guidelines governing research involving human participants.

## Results

The study population comprised 104 women undergoing repeat cesarean section, with the majority aged 26-30 years (n=44, 42.3%), followed by 31-35 years (n=38, 36.5%). Socioeconomic status was distributed as middle class in 45 participants (43.3%), low class in 36 (34.6%), and high class in 23 (22.1%). Regarding the type of previous cesarean section, 68 women (65.4%) had undergone emergency procedures, while 36 (34.6%) had elective cesareans. Comorbidities included hypothyroidism in 26 women (25.0%), GDM in 24 (23.1%), hypertensive disorders in 25 (24.03%), anemia in 20 (19.23%), and 33 women (31.7%) had no comorbid conditions. The indications for the previous cesarean were varied, with fetal distress accounting for 25 cases (24.04%), CPD in 19 (18.27%), cervical dystocia in 16 (15.38%), failed induction (medical) in 12 (11.54%), second stage arrest of labor in eight (7.69%), failed induction with PROM in nine (8.65%), macrosomia in six (5.77%), malpresentation in six (5.77%), and more than one previous cesarean in three cases (2.88%) (Table 1).

**Table 1:** Demographic and clinical characteristics of study participants (n=104) *Modified BG Prasad Scale The data is represented as n(%)

Variable	Total; n (%)
Age Group (years)	
21–25	18 (17.3)
26–30	44 (42.3)
31–35	38 (36.5)
>35	4 (3.8)
Socioeconomic Class*	
High	23 (22.1)
Middle	45 (43.3)
Low	36 (34.6)
Type of Previous CS	
Elective	36 (34.6)
Emergency	68 (65.4)
Comorbidity	
Anemia	20 (19.23)
Hypertensive disorders	25 (24.03)
Gestational diabetes mellitus	24 (23.1)
Hypothyroid	26 (25.0)
Nil	33 (31.7)
Indication of Previous CS	
Fetal distress	25 (24.04)
Failed induction (medical)	12 (11.54)
Failed induction w/ PROM	9 (8.65)
>1 previous CS	3 (2.88)
CPD	19 (18.27)
Macrosomia	6 (5.77)
Second stage arrest of labour	8 (7.69)
Cervical dystocia	16 (15.38)
Malpresentation	6 (5.77)

**Figure 1:**
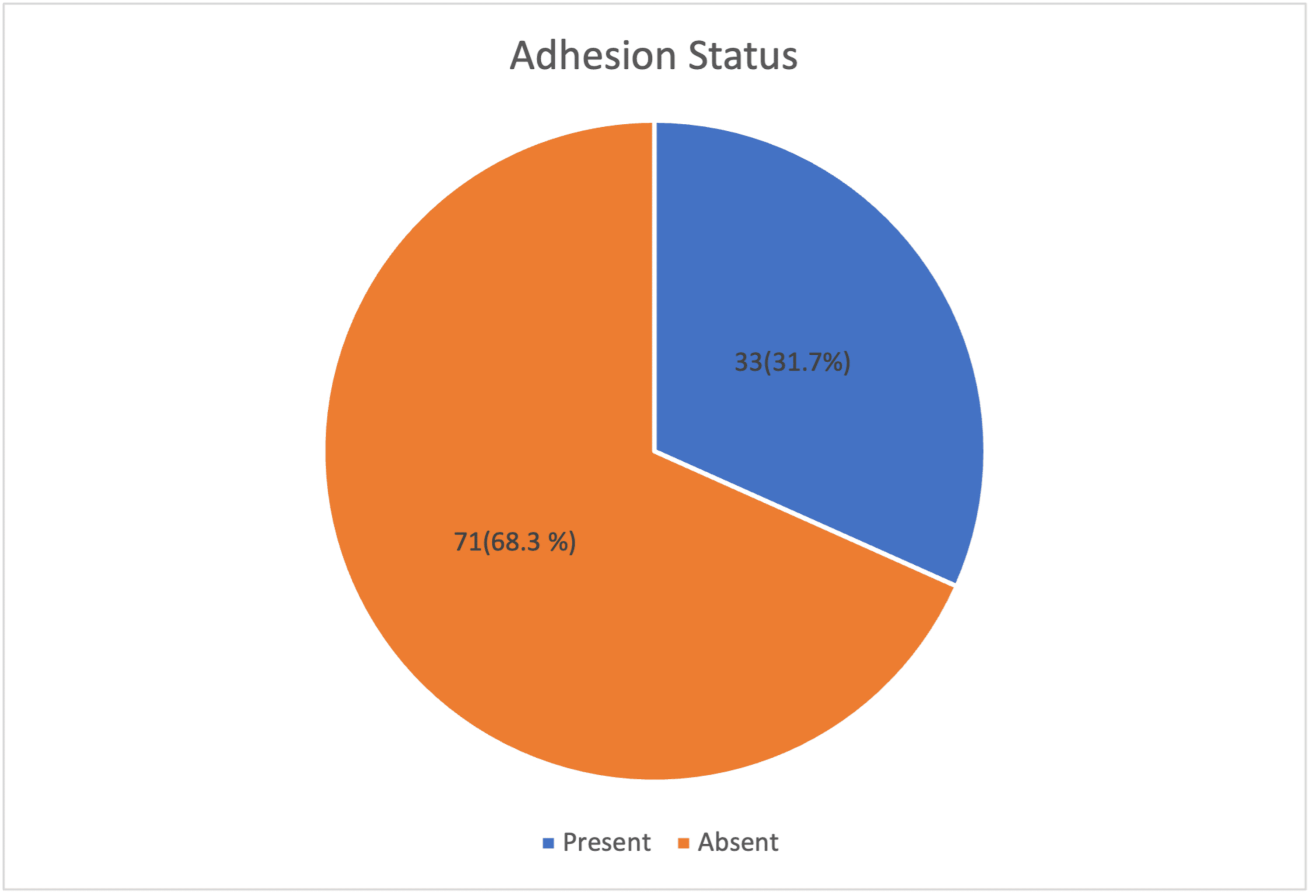
Distribution of intra-abdominal adhesions in repeat cesarean sections among the study participants (n=104) The data is represented as n(%)

In the present study, out of a total of 104 women who underwent repeat cesarean section, intra-abdominal adhesions were observed in 33 patients, accounting for 31.7% (95% CI: 23.2%-41.2%) of the study population. The remaining 71 women (68.3%; 95% CI: 58.8%-76.8%) showed no evidence of adhesion formation during the surgical procedure. This distribution indicates that nearly one-third of women with a history of previous cesarean deliveries developed postoperative adhesions, highlighting the considerable prevalence of this complication in repeat cesarean sections (Figure 1).

Among the 33 women with intra-abdominal adhesions, the most common site was between the omentum and abdominal fascia (n=11, 33.3%), followed by adhesions between the uterus and omentum (n=10, 30.3%), uterus and abdominal wall/fascia (n=7, 21.2%), and uterus and bladder (n=4, 12.1%). Only one patient (3.0%) had bowel involvement. Most adhesions were flimsy (n=27, 81.8%) and measured less than 3 cm in 16 cases (48.5%), while 11 (33.3%) were 3-6 cm and 6 (18.2%) exceeded 6 cm (Table 2).

**Table 2:** Distribution of adhesion site and severity among the study participants (n=33) The data is represented as n(%)

Adhesion Site and Severity	Total; n (%)	Flimsy; n (%)	Dense; n (%)	<3 cm; n (%)	3–6 cm; n (%)	>6 cm; n (%)
Uterus and bladder	4 (12.1)	3 (9.1)	1 (3.0)	4 (12.1)	0	0
Uterus and abdominal wall/fascia	7 (21.2)	5 (15.2)	2 (6.1)	6 (18.2)	1 (3.0)	0
Uterus and omentum	10 (30.3)	9 (27.3)	1 (3.0)	3 (9.1)	4 (12.1)	3 (9.1)
Omentum and abdominal fascia	11 (33.3)	9 (27.3)	2 (6.1)	3 (9.1)	5 (15.2)	3 (9.1)
Bowel	1 (3.0)	1 (3.0)	0	0	0	1 (3.0)
Total	33 (100)	27 (81.8)	6 (18.2)	16 (48.5)	11 (33.3)	6 (18.2)

**Figure 2:**
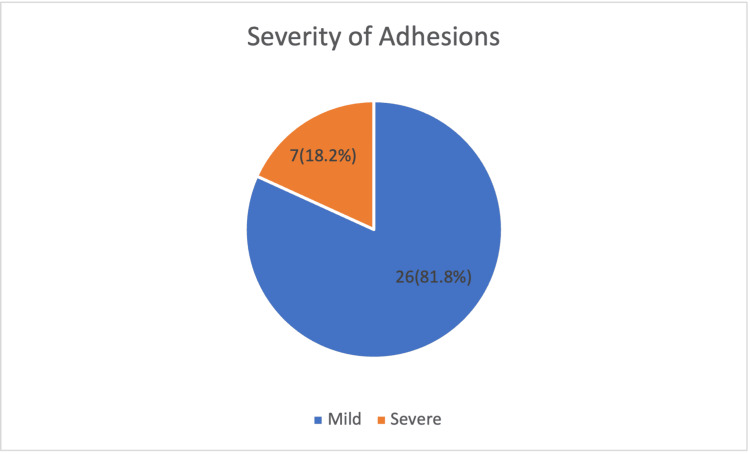
Severity of adhesions based on composite score (Classification according to Tulandi and Lyell) among study participants (n=33) The data is represented as n(%)

Figure 2 shows adhesion type according to the Tulandi and Lyell composite score; 27 (81.8%) cases were classified as having mild adhesions (score <16), while 6 (18.2%) were categorized as severe (score ≥16). This reflects that although adhesions were frequently observed in repeat cesarean sections, the majority were of lower severity, with fewer cases presenting significant operative challenges.

In the study population, adhesions were observed in 33 women during repeat cesarean section. Age-wise, adhesions were most common in women aged 26-30 years (n=17, 51.5%), followed by 21-25 years (n=8, 24.2%), 31-35 years (n=6, 18.2%), and >35 years (n=2, 6.1%), though this was not statistically significant (χ² = 6.16, p = 0.061). Socioeconomic status influenced adhesion formation, with the highest frequency in the low-income group (n=17, 51.5%) compared to middle (n=10, 30.3%) and high (n=6, 18.2%) classes (χ² = 6.21, p = 0.045). Adhesions were significantly more frequent after emergency cesarean sections (n=27, 81.8%) than elective procedures (n=6, 18.2%) (χ² = 5.41, p = 0.02). Among comorbidities, GDM was strongly associated with adhesions (n=16, 48.5% vs 8, 11.3%; χ² = 17.02, p < 0.001), while anemia (n=10, 30.3%; χ² = 3.84, p = 0.05) and absence of comorbidities (n=6, 18.2%; χ² = 4.73, p = 0.03) were also significant. Indications for previous cesarean such as second stage arrest of labor (χ² = 6.51, p = 0.02), cervical dystocia (χ² = 5.21, p = 0.022), failed induction with PROM (χ² = 5.62, p = 0.018), and fetal distress (χ² = 5.94, p = 0.015) were significantly associated with adhesion formation, whereas other indications including CPD, macrosomia, >1 previous CS, and malpresentation were not significant (Table 3).

**Table 3:** Association of socio-demographic and clinical characteristics with adhesions among the study participants (n=104) * Chi-square/Fischer exact test p-value <0.05 is statistically significant ^#^Modified BG Prasad scale.

Variable	Adhesions; n (%)	No Adhesions; n (%)	χ²	p-value
Age Group (years)				
21–25	8 (24.2)	10 (14.1)	6.16	0.061
26–30	17 (51.5)	27 (38.0)		
31–35	6 (18.2)	32 (45.1)		
>35	2 (6.1)	2 (2.8)		
Socioeconomic Class^#^				
High	6 (18.2)	17 (23.9)	6.21	0.045*
Middle	10 (30.3)	35 (49.3)		
Low	17 (51.5)	19 (26.8)		
Type of Previous CS				
Elective	6 (18.2)	30 (42.3)	5.41	0.02*
Emergency	27 (81.8)	41 (57.7)		
Comorbidity				
Anemia	10 (30.3)	10 (14.08)	3.84	0.05*
Hypertensive disorders	5 (15.15)	15 (21.13)	2.56	0.107
Gestational diabetes mellitus	16 (48.48)	8 (11.26)	17.02	<0.001**
Hypothyroid	6 (18.18)	10 (14.08)	0.29	0.589
Nil	6 (18.18)	28 (38.03)	4.73	0.03*
Indication of Previous CS				
Fetal distress	3 (9.09)	22 (31.0)	5.94	0.015*
Failed induction medical	2 (6.06)	10 (14.09)	1.45	0.23
Failed induction w/ PROM	6 (18.18)	3 (4.23)	5.62	0.018*
>1 previous CS	2 (6.06)	1 (1.41)	1.93	0.164
CPD	3 (9.09)	16 (22.54)	2.74	0.098
Macrosomia	1 (3.03)	5 (7.04)	0.68	0.41
Second stage arrest of labour	6 (18.18)	2 (2.82)	6.51	0.02*
Cervical dystocia	9 (27.27)	7 (9.86)	5.21	0.022*
Malpresentation	1 (3.03)	5 (7.04)	0.68	0.41

The comparison of surgical outcomes between women with and without adhesions demonstrated in Table 4, signifies that both blood loss and operation time were significantly higher in the presence of adhesions. Women with adhesions had a mean blood loss of 490.3 ± 127.4 ml compared to 433.9 ± 120.6 ml in those without adhesions, with a t-value of 2.17 (p = 0.03), indicating a statistically significant difference. Similarly, the mean operation time was markedly prolonged in the adhesion group at 67.3 ± 10.9 minutes versus 45.9 ± 7.6 minutes in the non-adhesion group, with a t-value of 9.12 (p < 0.001), representing a highly significant increase.

**Table 4:** Association of surgical outcomes with and without adhesions among study participants (n=104) * Independent sample t-test p-value <0.05 is statistically significant

Parameter	Adhesion (Mean ± SD)	No Adhesion (Mean ± SD)	t-value	p-value
Blood loss (ml)	490.3 ± 127.4	433.9 ± 120.6	2.17	0.03*
Operation time (min)	67.3 ± 10.9	45.9 ± 7.6	9.12	<0.001**

## Discussion

Intra-abdominal adhesions remain one of the most clinically significant sequelae following repeat cesarean sections, impacting both surgical outcomes and maternal morbidity [3,4]. These fibrous bands can cause operative challenges, increase the risk of visceral injury, prolong operative time, and contribute to higher blood loss during surgery. The prevalence of adhesions in our cohort was 31.7%, which aligns with rates reported in similar studies evaluating repeat cesarean deliveries [5,6]. This prevalence is somewhat lower than the 46% reported in certain Indian studies [14,15] and the 41% documented in Nepal [16], but notably higher than figures reported in Western populations, where adhesion-reducing surgical techniques are more consistently applied [17,18]. The variation in adhesion rates across regions may reflect differences in surgical practice, perioperative care protocols, patient characteristics, and institutional resources.

In examining demographic factors, we found that adhesions were most commonly observed among women aged 26-30 years. However, statistical analysis indicated that maternal age was not significantly associated with adhesion formation. This finding is consistent with prior observations by Lyell et al., who reported that age alone does not appear to be an independent risk factor for adhesion development [17]. The lack of association suggests that other factors, such as surgical technique, number of prior cesareans, and comorbidities, likely play a more critical role than maternal age in determining adhesion risk.

Socioeconomic status, however, demonstrated a significant association with adhesion formation. In our study, more than half of the women with adhesions belonged to the low socioeconomic class. These findings are consistent with observations by Nuamah et al., where lower socioeconomic status was linked to higher adhesion rates [19]. The underlying mechanisms may include poorer nutritional status, limited access to timely antenatal and postnatal care, and a higher likelihood of perioperative infections that predispose to adhesion formation [20,21]. Socioeconomic disparities may also reflect differences in access to high-quality surgical care, adherence to aseptic protocols, and availability of minimally invasive surgical techniques, which collectively influence adhesion risk.

A critical surgical factor associated with adhesion formation was the type of previous cesarean. In our study, 81.8% of adhesions occurred following emergency cesarean sections, compared to 18.2% following elective procedures. This observation parallels the findings of Jain et al., Pokhrel et al., and Elkoumy et al., who noted that emergency cesareans are more likely to result in adhesions due to the unplanned nature of the surgery, suboptimal operative conditions, and increased tissue trauma [5,16,22]. In emergency scenarios, limited time for careful dissection, increased intra-abdominal inflammation, and urgent fetal extraction contribute to heightened fibrotic responses and subsequent adhesion formation [23]. This emphasizes the importance of meticulous surgical technique and careful tissue handling, even in time-sensitive situations.

Comorbidities also influenced adhesion development in our cohort. GDM showed a strong association with adhesions (48.48% vs 11.26%, p < 0.001), which is consistent with prior studies indicating that hyperglycemia promotes excessive fibrotic response and impaired tissue healing [5,24]. Anemia was also significantly associated with adhesion formation (30.3% vs 14.08%, p = 0.05), likely due to impaired oxygen delivery and delayed wound healing. Interestingly, other comorbidities, such as hypertensive disorders (15.15% vs 21.13%; p = 0.107) and hypothyroidism (18.18% vs 14.08%; p = 0.589), did not show significant associations, which aligns with prior observations by Pokhrel et al. and Tulandi et al., where these conditions were not independent predictors of adhesion formation [16,24]. Furthermore, women without any comorbidity had a lower association for risk of adhesions compared to those with underlying health conditions, reinforcing the role of systemic health in post-surgical outcomes (18.18% vs 38.03%, p = 0.03).

The indication for the previous cesarean also appeared to influence adhesion development. Women with prior cesareans for cervical dystocia, second-stage arrest of labor, and failed induction with PROM had significantly higher rates of adhesions, while other indications, such as fetal macrosomia, malpresentation, and CPD, did not show a significant impact. These findings suggest that labor complications and difficult delivery conditions may increase tissue trauma, trigger an inflammatory response, and promote adhesion formation, a trend similarly reported by Lyell et al. and Tulandi et al. [17,25]. Conversely, cesarean sections performed for fetal distress in our study showed a lower proportion of adhesions, likely due to relatively straightforward surgical procedures without prolonged labor.

Our study confirmed that adhesions significantly affect operative outcomes. Women with adhesions had higher intraoperative blood loss (490.3 ± 127.43 ml vs 433.94 ± 120.61 ml, p = 0.03) and longer operative times (67.25 ± 10.91 min vs 45.93 ± 7.62 min, p < 0.001) compared to those without adhesions. These findings are consistent with prior research by Lyell et al. and Nuamah et al., which demonstrated that adhesions complicate repeat surgeries, increasing operative morbidity and risk of visceral injury [18,20]. Extended operative time and increased blood loss underscore the clinical relevance of adhesions in surgical planning and highlight the need for preventive measures during initial cesarean sections.

Regarding adhesion distribution, the omentum and abdominal fascia were the most commonly involved sites, followed by the uterus and omentum. Most adhesions were flimsy (81.8%) and small (<3 cm), although dense adhesions were predominantly noted at the uterus-abdominal wall interface, a pattern previously described by studies [26,27]. The predominance of flimsy adhesions is reassuring in terms of surgical manageability, but dense adhesions at critical sites increase the risk of bladder and bowel injury during repeat surgeries, emphasizing the importance of careful dissection and adherence to surgical safety protocols [4,5].

Limitations of our study include its single-center design and relatively small sample size, which may limit generalizability. Additionally, we did not evaluate long-term postoperative complications or fertility outcomes related to adhesions. The study relied on intraoperative assessment, which is inherently subjective, although we used a standardized scoring system (Tulandi and Lyell composite adhesion score) to mitigate variability. Regression analysis was also not performed to adjust for confounders, as not all variables were captured. Further multicenter studies with larger sample sizes are warranted to confirm these findings and explore preventive strategies.

Implications of our study are significant for clinical practice. Awareness of adhesion risk factors, including emergency cesarean, low socioeconomic status, and comorbidities such as GDM and anemia, can inform preoperative counseling and risk stratification. Emphasis on meticulous surgical technique, minimal tissue trauma, and adhesion-reduction strategies during cesarean section may help decrease postoperative complications in subsequent deliveries. Additionally, healthcare policy should consider targeted interventions for high-risk populations to reduce adhesion-related morbidity and optimize maternal outcomes.

## Conclusions

In conclusion, this study highlights that intra-abdominal adhesions are a common finding among women undergoing repeat cesarean sections, with a prevalence of 31.7%. Adhesion formation was more frequent following emergency cesarean deliveries and among women of lower socioeconomic status, as well as those with comorbid conditions such as anemia and GDM. The presence of adhesions was associated with increased operative time, greater intraoperative blood loss, and longer operative time, reflecting their impact on surgical complexity and maternal morbidity. Most adhesions were mild and flimsy, commonly involving the omentum and abdominal fascia, although dense adhesions at critical sites posed higher intraoperative risks. These findings underscore the importance of preventive measures - such as optimizing maternal health, minimizing surgical trauma, and adhering to meticulous operative techniques - to reduce adhesion formation and improve outcomes in repeat cesarean sections.
